# Does Diversity Climate Matter? The More Respected I Feel, the Better I Can Do: Unravelling the Mechanisms Enabling Employees’ Extra-Role Performance

**DOI:** 10.3390/bs14121164

**Published:** 2024-12-05

**Authors:** Belachew Kassahun Ayele, Wenbing Wu, Chong Chen

**Affiliations:** School of Economics and Management, Beijing Jiaotong University, Beijing 100044, China; wbwu@bjtu.edu.cn (W.W.); 20113079@bjtu.edu.cn (C.C.)

**Keywords:** diversity climate, thriving at work, workplace belongingness, leaders’ positive affective presence, extra-role performance

## Abstract

The current study examined how a diversity climate promotes employees’ extra-role performance and how the roles of workplace belongingness and thriving at work mediate this effect. Furthermore, this study investigated the sequential mediation effect of workplace belongingness and thriving at work in the aforesaid relationship. This study also tested the leaders’ positive affective presence as a moderator in the effect of diversity climate on extra-role performance. This study incorporated the self-determination theory to forge theoretical connections. As a result, a three-wave survey consisting of 349 employees revealed support that a diversity climate has a positive impact on employees’ extra-role performance, both directly and indirectly, through workplace belongingness and thriving at work. Meanwhile, leaders’ positive affective presence strengthens the diversity climate’s influence on workplace belongingness. Our findings supported all proposed hypotheses. Finally, this study discusses the theoretical and practical contributions of the results.

## 1. Introduction

In today’s ever-changing business landscape, the diversity climate continues to play a critical role in ensuring organizations’ survival. Organizations cultivate a diversity climate with the expectation of fostering improved employee performance [1,2]. Empirically, the literature has reported that the diversity climate has an impact at personal and organizational levels [3]. Indeed, the diversity climate is not surprising, as it provides employees with a sense of respect and value in the workplace. An effective diversity climate can be considered as the establishment and maintenance of an inclusive workplace devoid of discrimination [4] and has the potential to improve employees’ favorable work attitudes and behaviors. Conceptually, a diversity climate can have crucial effects on individuals, potentially leading to favorable outcomes for organizations, such as enhanced innovation, service, and safety [5,6]. By actively operationalizing the diversity climate policies and practices, organizations can nurture a supportive working environment, enabling employees to induce behaviors that benefit the organization. Particularly, in human-dominated organizations such as hotels, a diversity climate can fuel employees’ service-oriented motivations to exhibit innovative behavior and operational performance [7]. However, the hospitality literature acknowledges the importance of diversity climate and management [7,8], yet it neglects diversity climate as a key factor in employees extra-role performance. Given the importance of diversity climate through effective policies of equity and inclusion [2], it is important to understand how diversity climate influences employees’ extra-role performance.

An extensive body of literature has shown that diversity climate plays a catalyst role in garnering a high standing of firm effectiveness [9], facilitating learning among employees [10], enhancing employees’ job satisfaction [11], fostering team creativity [12], enhancing employees’ intention to stay [13], and fostering tactical knowledge sharing between employees [14]. This is because an increased pro-diversity climate environment positively influences the diversity of employees while mitigating the likelihood of misunderstandings and anxiety occurring among employees at the workplace [15,16]. In short, a diversity climate is likely to trigger high employee motivation by them being valued and supported, which in turn may motivate them to tackle obstacles and barriers to achieve organizational benefits. Such empirical evidence points that we should investigate how a diversity climate exerts a significant influence on employees’ extra-role performance.

Our investigation of the possible positive effects of how and when a diversity climate influences employees has also been inspired by the previous research on the positive effects of organizational diversity climate factors, including on employees’ in-role performance [1,17], organizational identification [18], and organization citizenship behavior [19]. The investigations highlighted how the diversity climate adopted by an organization becomes an important factor that affects the key results of the organization. From the above context, diversity climate as a formal structure fosters and values diversity [20], leading the organizational environment to positively influence employees’ feelings [21]. If organizations build fair and beneficiary treatment, this may lead employees to reciprocate the benefits of the organization [22], such as delivering extra-role performance [23]. Therefore, there is a need to exhibit the potential link between a diversity climate and favorable work for organizations (i.e., employee extra-role performance). Employees’ extra-role performance is considered a proactive behavior that initiates them to perform beyond role prescriptions [24]. Rescalvo-Martin et al. [25] pointed out that extra-role performance may pose an advantage to service firms in establishing organizational favor and enhancing their customer service. This is because service work at service firms frequently encompasses unforeseeable and thought-provoking circumstances, necessitating employees to transcend their prescribed roles to meet the challenging demands of customers [25]. Given the prominence of extra-role performance [26], it is surprising that there has been little research on diversity climate–extra-role performance linkage [19].

To address this issue, we adopt self-determination [27,28] as a theoretical lens for comprehending how and when a diversity climate may enhance employees’ motivational reactions, thereby promoting extra-role performance. Previous literature has focused on employee performance-related constructs as a catalyst of self-determination in organizations [27]. Exhibiting the relative significance of a diversity climate from the context of the self-determination theory is important to provide a much-needed novel perspective. Diversity climate is employees’ perception of an organization’s effort to promote an inclusive working place for employees with a diverse background [20]. The self-determination theory explains how a working environment characterized by a diversity climate may be perceived by employees as a potential motivator, thereby becoming a target of self-determination [27]. This perception in employees eventually contributes to seeing an organization’s diversity climate as non-discriminatory and fair in its social structure, leads them to actively participate in the organization’s basic structure, and promotes their social cohesion, which fosters their extra-role performance.

We propose to investigate whether workplace belongingness [29] and thriving at work [30] mediate the relationship between diversity climate and employees’ extra-role performance, both of which hold significant importance in the role of the self-determination theory (self-identification). Exploring such effects is theoretically important because it paints a complete picture of the diversity climate by investigating the situations under which employees’ work belongingness and thriving at work are facilitated. Workplace belongingness focuses on employee evaluations of the feelings of others within the organization [31]. Conversely, thriving at work refers to the conditions in which individuals feel enjoyment and vigor from their work and continuously learn, which provides the basis for applying innovative knowledge and competencies to enhance self-development [32]. Thus, there is promise in exploring how and when an organizational effort to promote a diversity climate affects employees’ attitudes toward their workplace, which are important for extra-role performance. This is because a diversity climate tends to influence employees’ workplace belongingness and thriving at work through its proximal motivational state.

**Figure 1:**
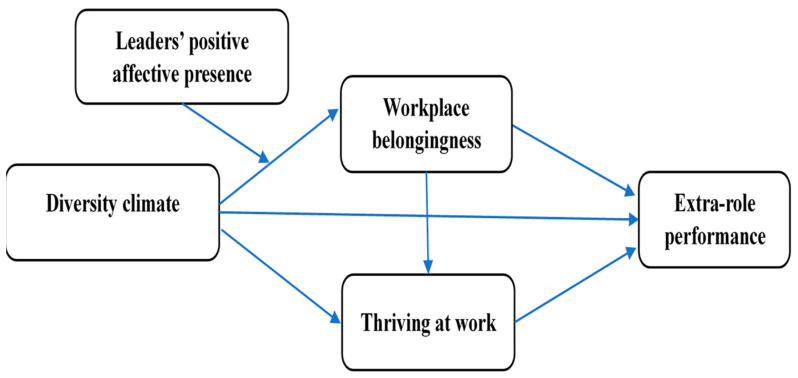
Conceptual model.

We further explore the leaders’ positive affective presence and their influence on employees’ feelings [33,34], moderating the nexus between diversity climate and workplace belongingness. According to Jiang et al. [33], leaders’ positive affective presence is defined as leaders’ behaviors intended to motivate employees and, over time, establish and maintain interpersonal relationships. In brief, a leader’s positive affective presence is most likely to evoke feelings of happiness, enthusiasm, and inspiration in their employees [35]. Extant literature on leader affective presence suggests that a leader’s positive affective presence provides abundant opportunities for employees to exhibit a greater propensity for flexibility, forward thinking, and motivation [34,35,36]. Drawing on the self-determination theory, we posit that a working environment having a positive leader’s affective presence plays a particular role in signaling respect, reliability, and value to employees, which ultimately cultivates employees’ workplace belongingness. Such a working environment influences employees’ well-being by enhancing or hindering their fundamental needs. Moreover, to build employees’ sense of belongingness at work, leaders’ positive affective presence often necessitates an increased interaction between employees and their leaders [37], which could fuel employees’ energy at work. See Figure 1 for the theoretical model.

Overall, the current study makes contributions in several ways. First, we link the diversity climate to employees’ extra-role performance to examine the possible influence exerted by the organizational environment and prove how a diversity climate elicits extra-role performance, thereby filling the void and contributing to the literature. Previous empirical work has made significant progress in determining how a diversity climate exerts beneficial effects on employees’ in-role performance [17,38]. This study contributes by examining the overlooked antecedents of extra-role performance, which are central to the focus of the study. In doing so, this article demonstrates the broad application of a diversity climate in the hospitality setting. Second, this study is the first to study the mediating roles of workplace belongingness and thriving at work to ascertain which are inherently more operative in promoting employees’ behavior and, therefore, affect their extra-role performance. Thus, the current study utilized mediating factors in the “diversity climate–extra-role performance” linkage, which is different from previous studies. Finally, we provide a fine-grained understanding of how leaders’ positive affective presence moderates the effect of diversity climate on workplace belongingness.

## 2. Theoretical Background and Hypothesis

### 2.1. The Effect of Diversity Climate on Extra-Role Performance

Diversity climate is the organizational environment that provides employees with a feeling of respect and value in the workplace. Dwertmann et al. [20] defined the diversity climate as the employees’ perceptions of the effectiveness of diversity-related activities in supporting and valuing a diverse workforce within an organization, which contributes positively to heightening the employees’ sense of inclusion [39]. As conceptualized, diversity climate matters to all employees [39] because every employee needs to be valued and treated fairly in the organization. In such organizations, employees are integral parts of the organization’s basic structure and strongly exhibit their full potential [40]. It is therefore reasoned that such incorporation into the organizational basic structure could help employees develop a strong ambition to exhibit extra-role performance aiming to elevate customer satisfaction. After all, extra-role performance is an instant behavior aiming at satisfying customers [41]. Moreover, an organization that encourages diversity through its diversity management promotes the well-being of employees by being included and valued [22]. They, therefore, see the promising organization’s goal in a way that motivates employees to focus on a set of proactive desired behaviors that contribute to the attainment of service quality [10], such as extra-role performance to meet customer demands.

Moreover, for the service sector, extra-role is linked with employees’ commitment to their customers and a higher level of the service process [42]. In this context, we expect that a positive perception of a diversity climate among employees can result in persistent service behavior at the workplace, eventually enhancing a memorable customer service experience [25]. Previous literature has reported that a diversity climate motivates employees to exhibit increased employees’ job performance [17,38], intensifies employees’ innovative service behavior [7], and strengthens organizational citizenship behavior [19], but less emphasis has been placed on extra-role performance. Based on the self-determination theory [43], which discusses how psychological needs drive the motivation for different qualities, we argue that an organizational diversity climate, the one that makes employees feel valued and respected, is an important factor that may drive extra-role performance. Thus, employees tend to perform with higher motivation. As suggested by the self-determination theory, the working environment affects employees’ functioning by either filling or impeding their fundamental needs [27,43]. Therefore, organizations with a diversity climate maximize the positive perception of employees as being valued, respected, and not discriminated through the policies and practices incorporated in plausible diversity management [22]. Thus, employees tend to view their work environment as significant, which fosters extra-role performance. Accordingly,

**H1.**  *Diversity climate positively affects extra-role performance.*

### 2.2. The Effect of Diversity Climate on Workplace Belongingness and Thriving at Work

A diversity climate in an organization tends to assume homogeneity in employees’ perceptions. Organizations should take diversity initiatives that justify the special attention paid to employees’ productivity through perceptive and emotional states [44]. As organizations tend to promote inclusiveness and fairness through diversity-related policies and practices [45], employees place a high value on the organization’s promotion of diversity and the feeling of value and respect they expect from it [46]. Taken together, employees develop strong perceptions because the organizational environment generates uniform responses, regardless of their background [47]. Positive diversity-related initiatives in an organization can substantially stimulate employees’ positive emotions [48], and these emotions are dispersed when individuals affirm the validity of their positive feelings through exchanges with their colleagues [49]. Consequently, an organization in which all employees are valued and respected may lead to an elevated sense of workplace belongingness and thriving at the workplace among employees.

Workplace belongingness is a complex construct in which employees evaluate the feelings of others in an organization and use these evaluations to determine their own sense of importance [31]. In other words, it is believed that workplace belongingness improves employees’ feelings of respect, acceptance, and unwavering support from others in the organization where they work. Thus, workplace belongingness is the situation that motivates employees, which consequently enables them to create and uphold interpersonal relations effectively [29]. The diversity climate for employees is supposed to increase the employees’ workplace belongingness by leveraging the principles and main values of diversity [22]. Specifically, an increased diversity climate can substantially strengthen employees’ psychological bonds with organizations by being valued and respected, which provides a strong sense of workplace belongingness [50]. Therefore, the organizational climate characterized by a positive diversity climate where employees perceive the organization as inclusive and promoting equity [22] is an enabling environment that is likely going to convey perceptions of being valued and respected members of the organization (i.e., workplace belongingness).

On a different note, thriving at work is a situation where individuals get a sense of learning and a heightened feeling of vitality at work [30]. Learning is perceived as the acquisition of new things or knowledge, consequently helping employees perform job tasks [30], while vitality describes employees’ feeling of being energetic and active at work [32]. As employees spend more time on work-related tasks, a diversity climate and a diverse workforce in an organization become important. Employees in a diversity climate resemble multicultural teams that foster their thriving at work [51]. By cultivating a strong diversity climate, organizations can enhance employees’ thriving at work, leading to engagement, innovation, and resilience [52]. This is because a high level of diversity climate positively affects employee perception [21]. In the same vein, a positive diversity climate significantly influences the employees’ perception of their value and respect within the organization, thereby increasing their motivation at work. In effect, an organization that sets a high level of diversity climate yields tangible benefits such as commitment [38], and recent research revealed that a diversity climate and managing it well drives employees to be fully engaged at work [7]. Similarly, a diversity climate might facilitate employees’ thriving at work.

Building upon the self-determination theory, we presume diversity management initiatives are important to generate an environment of respect, trust, and acceptance that aids in reducing the detrimental effects of employees’ perceptions and experience. As such, employees generally behave according to their sense of self, thriving, and motivation, which significantly influences [53]. Following this argument, a diversity climate could catalyze workplace belongingness and foster thriving at work among employees in an organization. At the heart of the self-determination theory is the fact that individuals tend to assimilate an activity to varying extents in a manner that could promote the realization of their psychological needs [43], which helps to form motivations of different qualities. Put differently, when employees get maximum satisfaction from their work and meet inherent psychological needs, they are likely to consider their workplace as significant and be willing to take part in their activities [27]. Ultimately, this leads to cultivating a deep sense of workplace belongingness [29] and the highest motivational quality for work, which is employees’ thriving at work [30]. Thus, with the integration of the preceding arguments and evidence, a diversity climate can significantly contribute to thriving at work and workplace belongingness. Accordingly,

**H2a.**  *Diversity climate positively affects workplace belongingness.*

**H2b.**  *Diversity climate positively affects employees’ thriving at work.*

### 2.3. The Effect of Workplace Belongingness on Thriving at Work

Research has substantiated strong support for workplace belongingness to cultivate employees’ thriving at work [54,55]. For instance, the interpersonal and social relations dimension of belongingness leads employees to focus more on intimate interactions and attachments [29]. In that sense, workplace belongingness is conceived as the perception of employees of how they are respected, accepted, and valued by members within an organization [31]. A working environment characterized by a diversity climate emphasizes individual standing and value and stimulates employees’ workplace belongingness [14]. Therefore, we argue that when employees’ sense of belongingness at work is increased, it encourages them to have a higher level of learning and vitality at work (i.e., thriving). Drawing on the self-identification theory, employees’ feeling of being deeply connected to a workplace and a social class through interaction promotes employees’ emotions because of their intrinsic motivation driven by their positive connections at the workplace [27], and it may be inferred that the inputs of workplace belongingness lead employees to learning opportunities and developing skills dedicated to improving tasks. Accordingly,

**H3.**  *Workplace belongingness positively affects thriving at work.*

### 2.4. The Effect of Workplace Belongingness and Thriving at Work on Extra-Role Performance

Workplace belongingness is generally viewed as positive employees’ emotional attachment to their organization by making them feel appreciated and respected [29]. This is true when an organization obeys specific principles of endorsing, such as reflecting fairness, justice, and equality for employees of different backgrounds and abilities [56]. As a result, employees pay back these bases of fairness, justice, and equality through desired behavior, such as extra-role performance. Extra-role performance is a set of proactive employee behaviors that surpass prescribed work roles [57,58]. This could be because employees who have a substantial sense of belongingness might be inclined to benefit the organization by meeting customer demands and expectations. Thus, organizations that establish and execute the diversity climate serve as the foundation for workplace belongingness, which in turn determines employees’ extra-role performance.

Unlike workplace belongingness, thriving at work fosters positive employees’ psychological outcomes such as development, growth, and competency [30]. Particularly, thriving is simultaneously a feeling of learning and vitality, which is a valuable tool for employees at work [30,32]. Employees who exhibit higher levels of learning and vitality typically display thriving towards their job [30], while those lacking one of these essential components may experience less thriving at work [32]. Thriving employees feel the significance of their jobs in the workplace, which drives them to exhibit positive work outcomes [59]. According to a previous study, thriving at work acts as a stimulus, driving motivation among employees and enabling them to get involved in extra-role performance [60]. This indicates that employees’ thriving at work will increase their abilities, which consequently leads to improved productivity, efficiency, and performance [32,61]. Thus, thriving at work enables employees to take on extra tasks to deliver service to customers.

Grounded in the self-determination theory, we assert that workplace belongingness and thriving at work will trigger employees’ meaningful involvement in extra-role performance. According to the self-determination theory, when employees perceive fair fulfillment of their fundamental psychological needs, they tend to exhibit favorable work behavior [53] to the extent to which employees’ motivation is of the highest quality. Thus, workplace belongingness and thriving at work are the keys to extra-role performance. Integrating the self-determination theory and the literature on workplace belongingness, we posit that workplace belongingness can be heightened when employees perceive the organization as being highly accepted and valued in its diversity management policies and practices [14]. As a result, frontline employees are more likely to become optimistic, confident, and willing to achieve superior extra-role performance. Put differently, a high level of thriving at work resulting from a diversity climate may contribute to extra-role performance. This is because a diversity climate helps to increase employees’ understanding of the different values of otherness and creates a workplace environment that ensures trust and quality [62], which is an enabling environment for employees to thrive at work. Highly thriving employees, thus, view themselves as having confidence in their abilities [30], which enables them to cope with various customer conditions. A higher level of thriving at work holds substantial potential for extra-role performance [63]. Accordingly,

**H4a.**  *Employees’ workplace belongingness positively affects extra-role performance.*

**H4b.**  *Employees’ thriving at work positively affects extra-role performance.*

### 2.5. The Mediating Role of Workplace Belongingness and Thriving at Work

The diversity climate reflects how employees perceive their working environment as non-discriminatory and socially conscious of every employee [15]. However, this study aims to exhibit the relative role of workplace belongingness and thriving at work as mediators between diversity climate and extra-role performance. Organizations possess heterogeneous workforces with regard to ethnicity, gender, operational context, and any further distinguishing characteristics [64]; however, this does not mean that this usually brings substantial benefits [65]. It is asserted that the effort to diversity can substantially create unparalleled problems, such as misunderstandings and anxiety among employees [66]. An organizational environment characterized by a promoted diversity climate, therefore, remains an elusive goal in the maintenance of an inclusive workplace [64] that facilitates the making of strong bonds among employees. This may enhance an employee’s sense of belongingness and thriving at work, which could help them exhibit a higher level of extra-role performance.

We propose that the diversity climate relates to employees’ workplace belongingness and, in turn, has a positive consequence for extra-role performance. Workplace belongingness is distinctly identified as the perception of employees of how they are respected, accepted, and valued by members within an organization [31]. It can be witnessed that it energizes employees’ social and interpersonal relationships [29]. Empirical work has found that employees’ workplace belongingness increases in organizations where employees perceive the diversity climate as non-discriminatory and socially inclusive of all employees [14]. Besides, existing research has revealed that belongingness is likely impacted in light of organizational-related factors and outcomes [67]. Surprisingly, existing studies on diversity have failed to capture workplace belongingness as a mediating conduit in the diversity climate–extra-role performance linkage. However, workplace belongingness enables employees to foster their helping behaviors [67,68], ultimately leading to greater engagement at work. As such, a workplace with a favorable diversity climate could foster employees’ self-belongingness, thereby stimulating thriving at work and extra-role behavior.

In contrast, we propose and test that diversity climate impacts employees’ thriving at work and, subsequently, yields a beneficial consequence for extra-role performance. According to Boehm et al. [15], the diversity climate provides employees the transparency and evidence that their organization is dedicated to valuing diversity. However, employees’ assessment of the organization’s fairness, justice, and equality remains uncertain; employees drive meanings from the organization’s structure, allowing the diversity climate to infer their respect and support by the organization [69]. The diversity climate thus allows organizations to continually improve equal treatment and employee value within an organization [18]. Aligned with the argument of Boehm et al. [15], when an organization establishes and maintains an inclusive workplace, devoid of discrimination, the pro-diversity activity sends unequivocal signals to the workforces that they are included and supported [4]. These perceptions motivate or energize employees at work by feeling included and supported [70], such as thriving at work, as the case might be in the present study. This might be promoted when employees’ responses to fair treatment are linked to pro-diversity. In addition, empirical work has validated that thriving at work enables employees to increase extra-role performance at the workplace [63]. Moreover, prior empirical studies have found that thriving at work could become a critical element for the various employees’ performance, such as creative performance [71] and innovative behaviors [72]. Ultimately, drawing on these findings, the improved employees’ thriving at work resulting from a diversity climate enables employees to foster extra-role performance.

Moreover, the work environment influences employees, which substantially improves employee efficiency and well-being by optimizing the effect of fundamental psychological necessities [27,43]. The organizational environment, characterized by a diversity climate, provides employees with the value, support, and respect they need to perceive the organization as non-discriminatory and supportive [22], and it sparks positive changes in employees’ behavior at work. Thus, such an environment is likely to cultivate perceptions of being deeply respected and treasured members of the organization (i.e., workplace belongingness) and thrive employees at work. This is because a diversity climate improves self-improvement, which consequently improves confidence and extra-role behaviors [73]. This behavior is discretionary and encourages employees to perform activities beyond their regular roles [74]. Additionally, the inherent psychological needs become a stimulus for employees to scrutinize their work environment, showing their level of belongingness [29]. Thus, in organizations where a diversity climate communicates organizational goodwill towards all employees, it may become more important to them. This heightened importance may increase the thriving at work and workplace belongingness, resulting in the fostering of extra-role performance. Accordingly,

**H5a.**  *Diversity climate indirectly and positively impacts extra-role performance via workplace belongingness.*

**H5b.**  *Diversity climate indirectly and positively impacts thriving at work via workplace belongingness.*

**H5c.**  *Diversity climate indirectly and positively impacts extra-role performance via thriving at work.*

**H5d.**  *Workplace belongingness indirectly and positively impacts extra-role performance thriving at work.*

### 2.6. The Moderating Role of Leaders’ Positive Affective Presence

Leaders’ positive affective presence captures leaders’ traits that encourage employees to react in the same way across different times and situations [34,35]. It is highly purported to motivate employees to experience happiness, enthusiasm, and inspiration. The concept is considered to influence employees’ attitudes and emotions [75]. The main value of a leader’s positive affective presence for employees is unquestionably its capacity to direct individuals to be confident and feel comfortable and encourage employees to be effective in activities facing highly challenging and hard work [34,35,75]. Therefore, it is considered that an enabling environment with a positive diversity climate, where employees perceive inclusivity [22], is an enabling environment that is likely to have leaders’ positive affective presence. Such leaders might be playing a critical role in shaping the diversity climate that fosters an incisive and emotionally supportive organizational environment more than the conventional leadership model. In brief, leaders’ positive affective presence is most likely to evoke feelings of happiness and inspiration in employees [34]. Extant literature has found that leaders with a positive affective presence more significantly enable employees to proactively manage situations at the workplace [33,76], often granting employees optimism and confidence [33], which fosters employees’ strong sense of ownership at their workplace. To these employees, leaders’ positive affective presence signals that they are always valued, trusted, and appreciated [34]. A lower level of leaders’ positive affective presence, in contrast, may make employees feel worried and stressed about the work context [34]. Studies reported that leaders with a lower level of positive affective presence are linked to unfavorable employee performance, thereby boosting unpleasantness toward the job and turnover intentions [77]. This is true when diversity is not valued, which muddles employees with a lack of confidence and fear of engaging in certain behaviors and restricts feelings and self-expression [78]. To advance the literature on diversity climate, we posit leaders’ positive affective presence as an auspicious moderator to strengthen the relationship between diversity climate and employees’ workplace belongingness, as employees feel appreciated and respected at the workplace.

Based on the self-determination theory, employees’ motivation can vary depending on the fulfillment of their psychological needs [28]. The assumption is that employees possess an intrinsic propensity for favorable psychological growth, internalization, and well-being, which is augmented through strengthened interaction with their environment, thereby facilitating the realization of these inherent tendencies. Put differently, leaders’ affective presence provides employees with confidence through an effective diversity climate with non-discriminatory and fair policies and practices [19]. This makes employees develop a sense of self; employees tend to integrate aspects of their essence and forge connections with others [28], which could improve employees’ workplace belongingness. Accordingly,

**H6.**  *Leaders’ positive affective presence has a moderating role in connecting diversity climate with workplace belongingness such that the relationship is stronger when leaders’ positive affective presence is high.*

## 3. Methodology

### 3.1. Data Collection and Procedure

Data were obtained from frontline employees who worked in star-rated hotels situated in Addis Ababa, Ethiopia. To recruit the respondents, we first visited the star-rated hotels to elucidate the study’s essence and objective and to get permission from the respective executive managers. Subsequently, we received authorization from the human resources (HR) divisions of the star-rated hotels. Then, to ensure the confidentiality of the responses, we contacted the employees who held positions involving direct contact with customers and had an organizational tenure of more than one year. A unique code for each frontline employee was given to identify frontline employees. To reduce the potential effect of common method bias [79], we administered the survey in three waves, separated by three weeks. In particular, at wave 1, employees were instructed to provide demographic information and rate the diversity climate. At that time, 423 employees had completed the set of questionnaires. At wave 2, employees rated leaders’ positive affective presence, thriving at work, and workplace belongingness, resulting in an 89.59% response rate. At wave 3, the employees rated their levels of extra-role performance, representing a response rate of 92.08%.

Finally, we successfully obtained valid employee questionnaires for 349 of the original participants. Of the total 349 employees, the mean age was 31.461 years (SD = 7.070), and 54.2% were female. The majority of them had obtained a degree and above (54.5%), and they had an average of 4.739 years (SD = 1.459) of tenure in the organization.

The current study scrutinized the proposed model using Smart PLS 4 techniques. The reason for using Smart PLS 4 software in the present study is that PLS-SEM is powerful yet user-friendly, and it can better handle data sets with non-normal distributions as well as intricate research models, particularly those featuring moderating and mediating variables [80].

### 3.2. Measurement Instruments

We adopted the measurement scales from prior studies to evaluate key variables. All items, except demographic variables, were measured using well-established scales from 1, “strongly disagree”, to 5, “strongly agree”. We converted all items into Amharic (a widely spoken language in Ethiopia) from English in a strict way by using a language expert, and inconsistencies were verified by two bilingual experts.

We measured diversity climate using a scale made up of four items from McKay et al. [81]. A sample item is “The company maintains a diversity-friendly work environment”.

We measured leaders’ positive affective presence with three items adapted from Madrid et al. [34]. A sample item is “To what extent interacting with your team leader makes you feel inspired”.

We used the scale developed by Jena and Pradhan [82] to measure workplace belongingness, which has twelve items. A sample item is “I feel that there is a semblance between my organization and my own values and beliefs”.

Thriving at work was measured using an eight-item scale from Porath et al. [32]. A sample item is “I find myself learning often”.

Extra-role performance was measured using a scale of eight items adapted from Williams and Anderson [83]. A sample item is “I go out of way to help new employees”.

Furthermore, previous research has indicated a potential connection between extra-role performance and control variables [42,84]. Therefore, our study accounted for the influence of respondents’ age, gender, education level, and tenure in organizations.

## 4. Results

### 4.1. Measurement Model Evaluation

We evaluated the current study’s measurement model, following the steps to ensure that the data support the study’s hypotheses statistically. To test the measurement model, we followed the two-step procedures for validation of construct reliability and construct validity. We evaluated Cronbach’s alpha values and composite reliability (CR) values to validate the indicator reliability, as shown in Table 1. All values were greater than the prescribed threshold of 0.7 for Cronbach’s alpha and composite reliability (CR) [85], we confirm the construct reliability.

Subsequently, we analyzed the construct validity using the convergent and discriminant validity, as well as their significance level. To confirm the convergent validity of the measurement model, we evaluated the average variance extracted (AVE) and factor loadings. All our constructs had AVE values greater than the recommended cut-off of 0.5, and all indicators and dimension loadings in our model exceeded 0.6 [86], indicating convergent validity. To confirm the appropriateness of the construct discriminant validity, we evaluated the heterotrait–monotrait ratio, as presented in Table 2. All the HTMT values for constructs remained below 0.90 [87], indicating support for discriminant validity.

### 4.2. Structural Model Assessment

The statistical results obtained from the measurement model evaluation confirm that the model is empirically viable for further analysis. Table 3 also shows that the structural model fits by examining the independent variables to explain total variance (R^2^). Furthermore, the structural model fit for workplace belongingness, thriving at work, and extra-role performance yielded 0.373, 0.436, and 0.402, respectively. The current study thus exhibits good structural model fit. A value larger than 0.20 reflects that the model has a reasonable competency for analyzing the constructs [80]. To approximate the data for the proposed model, we evaluated SRMR using estimated and saturated models, as shown in Table 3. The values for saturated and estimated models were statistically significant and below 0.08 [80], conforming to both the saturated and estimated models that received support. As shown in Table 3 *t*-test values were greater than the minimum cut-off of 1.96 with a 95% confidence interval. In addition, we assessed $Q^{2}$ using blindfolding procedures to confirm whether the isolated part of the sample reproduces its value. As shown in Table 3, the results for workplace belongingness, thriving at work, and extra-role performance were 0.354, 0.360, and 0.252, respectively. The results satisfy the requirements of $Q^{2};$ as shown by the values, the model exhibited a significant fit to replicate a portion of the sample [87]. In summary, the measurement model is robust and meets all the preconditions.

### 4.3. Hypotheses Testing

To test the proposed hypothesis, we utilized a bootstrapping technique (two-tailed 5000 sub-samples) to confirm the consistency of the path coefficients. As reported in Table 3, diversity climate had a positive effect on extra-role performance (β = 0.204, *p* < 0.01), supporting H1. Diversity climate was positively related to workplace belongingness (β = 0.595, *p* < 0.001), supporting H2a. The positive relationship between diversity climate and thriving at work was significant (β = 0.409, *p* < 0.001), supporting H2b. Workplace belongingness unveiled a direct correlation with thriving at work (β = 0.332, *p* < 0.001), supporting H3. Workplace belongingness had a positive impact on extra-role performance (β = 0.361, *p* < 0.001), supporting H4a. Moreover, thriving at work had a direct and positive impact on extra-role performance (β = 0.167, *p* < 0.05), supporting H4b. Additionally, we found all the links between controlling variables and extra-role performance to be non-significant.

Next, we applied the 5000-bootstrapped samples technique with 95% bias-corrected CI to analyze the mediation effects. With this bootstrapping technique, we confirmed the indirect effects of diversity climate and workplace belongingness on thriving at work and extra-role performance (see Table 3). The indirect effects of diversity climate on extra-role performance and on thriving at work via workplace belongingness were significant (β = 0.215, *p* < 0.001, 95% CI [0.129, 0.309], and β = 0.197, *p* < 0.001, 95% CI [0.132, 0.278]). Thus, the result confirms the mediating effect of workplace belongingness, supporting hypotheses 5a and 5b.

Furthermore, thriving at work was a significant positive mediator between diversity climate and extra-role performance (β = 0.069, *p* < 0.05, 95% CI [0.013, 0.142]). Additionally, thriving at work played a substantial mediating role in the association between workplace belongingness and extra-role performance (β = 0.056, *p* < 0.05, 95% CI [0.011, 0.116]). Thus, the result confirms that thriving at work holds a mediating effect, supporting 5c and 5d.

**Figure 2:**
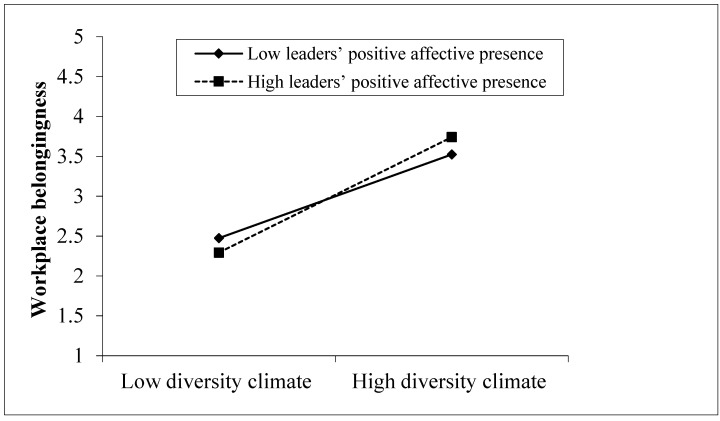
The moderating effect of leaders’ positive affective presence on the diversity climate–workplace belongingness relationship.

Finally, for moderation effects, we predicted that leaders’ positive affective presence strengthens the effect of a diversity climate on workplace belongingness (hypothesis 6). As presented in Table 3, the interaction of diversity climate with leaders’ positive affective presence on workplace belongingness was significant (β = 0.199, *p* < 0.001, 95% CI [0.111, 0.288]). Moreover, as displayed in Figure 2, the positive effect of diversity climate on workplace belongingness was stronger for employees with high leaders’ positive affective presence (β = 0.700, *p* < 0.001, 95% CI [0.589, 0.812]) than for those with low leaders’ positive affective presence (β = 0.350, *p* < 0.001, 95% CI [0.244, 0.457]). Therefore, the moderation analysis robustly strengthens the impact of diversity climate on employees’ workplace belongingness, supporting H6.

## 5. Discussion and Conclusions

The ever-evolving landscape of the hospitality industry requires employees who are prepared to go beyond the set of tasks to elevate customers’ pleasure levels [25]. More importantly, employees are considered active actors to maintain positive effects on customers in terms of satisfaction, thus increasing organizational profitability. The current study examined how a diversity climate promotes employees’ extra-role performance and how the roles of work belongingness and thriving at work mediate this effect. In line with expectations, a diversity climate was identified as being related constructively to extra-role performance. In that sense, employees value organizational initiatives to the extent to which organizations value, respect, and make employees part of the organization through diversity-related policies. The more they perceive the organization is eliminating discrimination at the workplace and that diversity-related policies are fair, the more it provides employees with clues that they are valued and respected (i.e., a diversity climate). Our study expands the general literature that has focused on reward and recognition, empowering leadership, leaders’ behavior, performance appraisal and performance feedback, high-performance HR practices, and job characteristics to understand employees’ extra-role performance [42,84,88,89].

### 5.1. Theoretical Implications

This study counts as the first to investigate workplace belongingness and thriving at work as pathways to link diversity climate with extra-role performance. The net effect of a diversity climate on workplace belongingness and thriving at work, along with its subsequent consequences, depends on how well employees perceive their working diversity climate. Besides, our findings highlight the urgency of creating a diversity climate to frame positive employees’ diversity perceptions because when employees are treated equally and without discrimination, they are likely to be more committed. Organizations implementing diversity-related policies and practices irrespective of employees’ differences will serve as precedents for employees’ extra-role performance. Furthermore, our findings agree with the previous findings of Gavino et al. [73] and Singh et al. [19], which found that organizational diversity climate initiatives and practices enhance employees’ extra-role performance at work.

Our findings illustrate that a diversity climate generates a unique contribution to employees’ belongingness at work. Organizations that adopt a working climate characterized by diversity improve employees’ sense of belongingness at work, but also a keen interest in it. It is significant for organizations to continually improve unwavering support since their support directly contributes to improving feelings of respect and acceptance among employees. Our findings complement the results obtained by previous researchers who discovered the impacts of a diversity climate. For example, the work of Enwereuzor [14] suggested that organizational working environment support for diversity is a key driver of employees’ workplace belongingness at work.

This study also proves that a diversity climate promotes employees’ thriving at work. In doing so, we increased our insights into how a diversity climate provides signals to employees that they are valued and motivated. Moreover, existing literature reported such a positive diversity climate drives employees’ thriving at work [90]. Furthermore, our finding suggests that a working environment characterized by a diversity climate can promote thriving at work and thus increase employees’ greater level of skills and experience.

Our findings indicate a strong correlation between workplace belongingness and thriving at work. The results suggest that a higher level of belongingness at work leads to thriving employees, with an optimistic state of mind motivating employees’ learning and vitality at the workplace. Empirically, Gkorezis et al. [54] and Al Riyami et al. [55] proposed that employees’ thriving at work can be improved through employees’ workplace belongingness. In view of our results, when employees’ workplace belongingness is high, it forces them to thrive at work by allowing greater learning and vitality.

Concerning workplace belongingness’ effect on extra-role performance, workplace belongingness exerts a positive and significant influence on employees’ extra-role performance. Our study suggests that a higher level of employees’ self-belongingness at work can positively improve their extra-role performance. These findings validate those of Kyei-Poku and Yang [91], indicating that employees tend to perform roles beyond the roles assigned when they experience an increased sense of belongingness.

The current study underscores that thriving employees at work are likely to maximize extra-role performance. When employees feel the significance of their jobs in the workplace, they are predisposed to exhibit high work outcomes [30,59]. This result concurs with the previous findings showing how employees’ thriving at work has the potential to motivate employees to display more extra-role behavior at work [63]. Because the nature of the work and social interaction determines employees’ behavior, these can lead individuals to exhibit favorable work behaviors and expand the boundaries of their assigned tasks [32].

Our work also reveals that workplace belongingness plays a vital role in mediating a diversity climate with extra-role performance. We empirically found that a higher standing of diversity climate in organizations contributes to the enhancement of employees’ extra-role performance by increasing their workplace belongingness. This study suggests that when employees have feelings of being included and valued in a diversity climate, this could develop their sense of belongingness and performance at the workplace. Therefore, the diversity climate can be even more crucial for employees’ workplace belongingness, thus, in turn, enhancing their extra-role performance. The current study offers a complementary explanation of the previous findings, suggesting that diversity climate is crucial to shaping tactical knowledge sharing [14] and that interactional fairness is crucial to promoting interpersonal citizenship behavior [67].

Furthermore, the current study has taken steps to reveal that a favorable diversity climate is a key to thriving employees through workplace belongingness. The previous literature, however, has not addressed the results. This study, thus, finds that the path that links a diversity climate to thriving at work is significant, thereby enhancing the effectiveness of employees. Accordingly, our research advances the existing body of literature on employees’ workplace belongingness; this study discovered a more fine-grained insight into workplace belongingness as a mediator in connecting diversity climate with thriving at work. This suggests that organizations with a pro-diversity climate appear to enhance employees’ workplace belongingness. Employees who often have relatively high standings of workplace belongingness are good at thriving at work and, thus, are more effective at performing extra-role behavior.

The current study provides evidence that thriving at work significantly mediates diversity climate to extra-role performance. As expected, the results reveal that employees with high perceptions of fairness in an organizational diversity climate are more able to cultivate the spirit of learning and vitality and promote their extra-role performance, thereby facilitating service delivery and meeting unpredicted customer demands. Literature has not recognized thriving at work as an important mediator in the diversity climate with employee’s extra-role performance, despite previous research providing support to the notion by showing the mediating role of thriving at work on contextual factors–affective organizational commitment linkage [92]. The findings of the present study are noteworthy, given that a competent organizational diversity climate is most likely to be perceived as fair and non-discriminatory by employees. Based on this logic, it allows employees to display desirable behavior by being thriving employees at work.

This study offers plausible evidence that thriving at work is an important mediator between workplace belongingness and extra-role performance. The results suggest that increased workplace belongingness appears to generate increased employees’ thriving at work, ultimately fostering extra-role performance. Although few studies have illustrated the self-work crafting to workplace belongingness and thriving at work relationship [55], the literature did not reveal that thriving at work had mediated the workplace belongingness–extra-role performance relationship. The current study, therefore, is a pioneer that provides initial insights into how thriving at work could mediate the relationships between diversity climate and extra-role performance.

Our empirical study regarded leaders’ positive affective presence as a moderator in the relationship between diversity climate and workplace belongingness. The results show the position of positive leaders’ affective presence to determine the employees’ workplace belongingness, facilitating greater identification, energy, and ultimately greater commitment. Though our study can be seen to concur with the earlier literature, suggesting that a better diversity climate in an organization gives rise to employees’ workplace belongingness [14], it adds to the body of literature by revealing that positive leaders’ presence enables employees to develop a high sense of self in the workplace.

Additionally, the current study draws from the self-determination theory as a consolidative lens [27,43] along with various previous literature, including diversity climate, positive leaders’ presence, workplace belongingness, and extra-role performance to look into how a diversity climate promotes extra-role performance by mediating the roles of workplace belongingness and thriving at work, thereby improving our insights about the interplay of these variables. This study additionally contributes to the general literature on diversity climate by adding an important construct of positive leaders’ affective presence to moderate the effect of diversity climate on employees’ workplace belongingness.

### 5.2. Practical Contributions

This study has several pragmatic implications for organizations intended to promote employees’ extra-role performance. It is, therefore, definitely important to recognize the diversity climate as it has motivational power for employees at the workplace. Our study expands organizational members’ understanding of the positive effects of a diversity climate on employees for extra-role performance. Considering the significance of a diversity climate in promoting employees’ extra-role performance, organizations should take action to establish an environment that emphasizes diversity and manages it well. Such an environment is more likely to be non-discriminatory and cultivate a higher sense of inclusion and support in employees regardless of their differences. Stress on diversity is often vital to the success of employees within an organization [64]. Indeed, managers could establish and maintain an inclusive workplace by upholding a collective identity devoid of discrimination, using symbolic management that cultivates an ongoing and constructive diversity climate for employees [4].

Hospitality managers should uphold the diversity climate that resonates significantly with the current work context and invest the optimal resources to avoid discrimination in areas that highlight individual differences. An effective diversity climate offers employees the necessary value and support through organizational policies and practices, allowing them to exhibit their extra-role performance. In general, studies suggest that organizations that prioritize promoting diversity initiatives and management when implementing human resource policies and practices improve employees’ organizational citizenship behavior [19]. This implication is particularly important to hospitality organizations that operate to address the diverse demands of customers.

Hospitality organizations should be aware of workplace belongingness and thriving as motivational factors at the workplace to elicit high extra-role performance among frontline employees. This implication is important because it highlights workplace belongingness and thriving at work as a crucial path by creating an organizational climate where employees feel accepted, respected, included, and supported to foster employees’ extra-role performance. Such a diversity climate stimulates inclusivity as the way forward at work; therefore, diversity policies become effective for employees [93]. Managers need to develop a working environment characterized by a diversity climate that triggers positive employee workplace belongingness and thriving at work.

As the findings in the current study suggest, positive leaders’ presence acts as a buffer to strengthen the influence of a diversity climate on workplace belongingness. The relationship is accentuated in conditions of high compared to low positive leaders’ affective presence. Hence, to trigger the favorable effect of a diversity climate, leaders should acknowledge sufficient concern and ongoing support to employees, as their positive affective presence can render the workplace belongingness of employees effective. Scholars have suggested that leaders’ positive affective presence can provide abundant feelings of contentment, passion, and inspiration for employees at work [34,35]. Such leaders can create a partnership with employees; thus, employees develop a sense of workplace belongingness.

Moreover, hotel managers should acknowledge the importance of creating and maintaining a diversity climate that conveys employee transparency and demonstrates the organization’s dedication to valuing diversity. An effective diversity climate is more effective when it is inclusive, targets all employees, and fosters collaboration among them [7,17,18]. Hotel managers should focus on creating a diversity climate that promotes diversity, equity, and inclusion through effective policies [7]. In doing so, it will serve as a valuable resource in employee development, playing a crucial role in promoting motivation, positive attitudes, and desirable behavior at work.

### 5.3. Limitations and Future Research

The current study appears to shed new light on the crucial paths that link diversity climate to employees’ extra-role performance (i.e., workplace belongingness and thriving at work) under a cross-sectional method. It also proves, for the first time, that leaders’ positive affective presence plays a high-standing moderator role in strengthening the effect of diversity climate on employees’ workplace belongingness. Such valuable insights point to the robustness of our findings, but we also acknowledge a set of limitations and provide ideas that the coming research could address. First, our research appears to test the proposed theoretical model using convenience samples obtained from employees. Though this method has obtained acceptance in hospitality research due to the sector’s unique features [94], we, therefore, may not generalize the findings of the study. Upcoming research could replicate our findings by employing a large sample of representatives for more comprehensive empirical insights. Second, this study adopted a cross-sectional method to analyze employees’ views at a specific moment in time, which may limit the study’s comprehensiveness as behavioral changes occur over time [95]. Future studies should adopt a longitudinal research approach to contrast our findings. Such an approach determines the longitudinal behavior variables over time and solves the causality issues. Third, the current study only considered data collection in the hotel sector, thus limiting generalization to different cultural settings. To validate our findings, future researchers could conduct research outside the hospitality sector. Fourth, the current study only considers age, gender, education level, and tenure in organizations as controlling variables. Future studies could consider nationality or ethnicity, using a larger, more representative sample to provide comprehensive empirical insights. Five, our study considered only workplace belongingness and thriving at work as mediators and the leader’s positive presence as a moderator; thus, future research could consider the exploration of other factors to broaden understanding of the impact of diversity climates. Therefore, upcoming research could consider potential mediators, such as team cohesion [96] and organizational identification [97], and moderators, such as psychological safety [98].

## Figures and Tables

**Table 1:** Validity and reliability of items.

Constructs	Items	Loading	α	CR	AVE
Diversity climate	DC1	0.773	0.803	0.871	0.629
	DC2	0.800			
	DC3	0.821			
	DC4	0.775			
Leaders’ positive affective presence	LAP1	0.817	0.823	0.89	0.729
	LAP2	0.837			
	LAP3	0.905			
Thriving at work	TW1	0.793	0.901	0.921	0.592
	TW2	0.776			
	TW3	0.706			
	TW4	0.797			
	TW5	0.825			
	TW6	0.774			
	TW7	0.768			
	TW8	0.709			
Workplace belongingness	WB1	0.784	0.935	0.944	0.584
	WB2	0.794			
	WB3	0.713			
	WB4	0.783			
	WB5	0.785			
	WB6	0.760			
	WB7	0.785			
	WB8	0.775			
	WB9	0.796			
	WB10	0.770			
	WB11	0.705			
	WB12	0.707			
Extra-role performance	ERP1	0.735	0.91	0.926	0.612
	ERP2	0.797			
	ERP3	0.757			
	ERP4	0.797			
	ERP5	0.791			
	ERP6	0.806			
	ERP7	0.794			
	ERP8	0.779			

**Table 2:** Heterotrait–monotrait ratio (HTMT), mean, and standard deviation.

Variables	1	2	3	4	5	6	7	8	9
1. Age									
2. Gender	0.025								
3. Education	0.149	0.037							
4. Tenure	0.054	0.064	0.529						
5. DC	0.029	0.046	0.112	0.111					
6. LPAP	0.130	0.048	0.045	0.034	0.153				
7. TW	0.049	0.038	0.032	0.045	0.697	0.142			
8. WB	0.065	0.047	0.049	0.055	0.667	0.074	0.621		
9. ERP	0.024	0.091	0.037	0.035	0.586	0.102	0.540	0.618	
Mean	31.461	1.540	2.520	4.739	3.595	3.455	3.400	3.509	3.644
Standard deviation	7.070	0.5	0.808	0.807	1.459	0.809	0.831	0.799	0.792

Note: *n*: 349; DC, diversity climate; LPAP, leaders’ positive affective presence; TW, thriving at work; WB, workplace belongingness; ERP, extra-role performance.

**Table 3:** Evaluation of the structural model.

Effects Type	Coefficient	*t*-Value	CI	$f^{2}$	$R^{2}$	$Q^{2}$
Direct effect						
Age→ERP	0.008 (0.843)	0.198	[−0.072, 0.089]	0.000		
Gender→ERP	0.151 (0.072)	1.796	[−0.014, 0.313]	0.009		
Education→ERP	0.001 (0.983)	0.022	[−0.086, 0.087]	0.000		
Tenure→ERP	0.055 (0.266)	1.113	[−0.044, 0.148]	0.004		
DC→ERP	0.204 ** (0.002)	3.030	[0.074, 0.336]	0.038		
DC→WB	0.595 *** (0.000)	13.626	[0.509, 0.677]	0.553		
DC→TW	0.409 *** (0.000)	8.173	[0.307, 0.503]	0.196		
WB→TW	0.332 *** (0.000)	6.144	[0.222, 0.435]	0.129		
WB→ERP	0.361 *** (0.000)	5.302	[0.221, 0.490]	0.127		
TW→ERP	0.167 * (0.023)	2.266	[0.031, 0.320]	0.026		
Moderation effect						
DC × LPAP→WB	0.199 ** (0.000)	4.413	[0.111, 0.288]	0.053		
Mediation effects						
DC→WB→ERP	0.215 *** (0.000)	4.738	[0.129, 0.309]			
DC→WB→TW	0.197 *** (0.000)	5.351	[0.132, 0.278]			
DC→TW→ERP	0.069 * (0.038)	2.077	[0.013, 0.142]			
WB→TW→ERP	0.056 * (0.04)	2.050	[0.011, 0.116]			
Endogenous variables						
WB					0.367	0.354
TW					0.436	0.360
ERP					0.402	0.252
Overall fit						
SRMR saturated model fit 0.056						
SRMR Estimated model fit 0.058						

Note: *n*: 349; * *p* < 0.05, ** *p* < 0.01 and *** *p* < 0.001; bootstrapping sample size 5000; DC, diversity climate; LPAP, leaders’ positive affective presence; TW, thriving at work; WB, workplace belongingness; ERP, extra-role performance.

## Data Availability

The raw data supporting the conclusions of this article will be made available by the authors, without undue reservation.
